# Cardioprotective Effect of Echinatin Against Ischemia/Reperfusion Injury: Involvement of Hippo/Yes-Associated Protein Signaling

**DOI:** 10.3389/fphar.2020.593225

**Published:** 2021-01-11

**Authors:** Jieting Niu, Yanguang Li, Xiang Song, Yunfeng Liu, Ying Li, Ya Li

**Affiliations:** ^1^ Department of Geriatrics, Cangzhou Central Hospital, Cangzhou, China; ^2^ Department of Thoracic Surgery, Cangzhou Central Hospital, Cangzhou, China; ^3^ Department of Cardiology, Cangzhou Central Hospital, Cangzhou, China

**Keywords:** echinatin, myocardial ischemia/reperfusion injury, hippo/yes kinase-associated protein signaling, caspase-3, lactate dehydrogenase

## Abstract

**Background:** Echinatin (Ech) has been reported to exert antioxidant and anti-inflammatory activities. In this study, we aimed to characterize the functional role of Ech in myocardial ischemic/reperfusion (MI/R) injury and elucidate its underlying mechanism of action.

**Method:** We established *in vivo* and *in vitro* models of MI/R injury to determine the effect of Ech on MI/R injury. Gene expression was examined using quantitative real-time polymerase chain reaction and western blotting. Myocardial infarction was assessed using tetrazolium chloride staining and the degree of myocardial injury was evaluated by measuring lactate dehydrogenase (LDH) and creatine kinase-myocardial band (CK-MB) levels. Cell apoptosis was detected using the terminal deoxynucleotidyl transfer-mediated dUTP nick end-labeling (TUNEL) assay. The viability of H9c2 cells was determined using Cell Counting Kit-8 assay.

**Results:** MI/R induced myocardial infarction, which was mitigated by Ech treatment. Moreover, Ech treatment resulted in a marked decline of LDH and CK-MB levels in the serum and myocardium of MI/R rats. Ech treatment also restrained cardiomyocyte apoptosis *in vivo* and *in vitro*, as evidenced by reduction in LDH release, the number of TUNEL-positive cells, and caspase-3 activity. Furthermore, Ech administration inhibited MI/R-induced activation of Hippo/Yes-associated protein signaling *in vivo* and *in vitro*, as indicated by inhibition of mammalian sterile 20-like protein kinase 1, large tumor suppressor one, and YAP phosphorylation and promotion of YAP nuclear translocation. However, silencing of YAP counteracted the protective effect of Ech on hypoxia/reoxygenation-induced myocardial injury *in vitro*.

**Conclusion:** Ech exerted its protective effect against MI/R injury at least partially by suppressing the Hippo/YAP signaling pathway, providing novel insights into the remission of MI/R injury.

## Introduction

Myocardial infarction, resulting from the interruption of blood supply, is one of the most frequent cardiovascular emergencies, which remains to be the major cause of mortality and disability worldwide (Thygesen et al., 2018). To date, restoration of blood flow in the infarcted coronary artery as soon as possible is considered as the most effective treatment for myocardial infarction (Anderson and Morrow, 2017). However, restoration of coronary circulation may also aggravate reversible myocardial ischemia injury and lead to irreversible myocardial injury, termed as myocardial ischemic/reperfusion (MI/R) injury (Ferrari et al., 2017). MI/R injury is associated with poor clinical outcomes in patients with myocardial infarction and causes multiple cardiac complications. MI/R is generally recognized as the serious pathophysiologic mechanism of myocardial infarction (Reed et al., 2017). In view of this, reducing MI/R injury is necessary to reduce the myocardial infarct size and improve myocardial function.

Among these, glycyrrhiza has attracted much attention from researchers in the last few years. It has been shown to exhibit multifarious pharmacological activities, such as anti-myocardial infarction, anti-tumor, and anti-viral capabilities (Ojha et al., 2013). *Glycyrrhiza* contains many kinds of bioactive constituents, such as isoliquiritigenin, glycyrrhetinic acid, and licochalcone D., which have protective effects against MI/R injury (Zhang et al., 2013; Wu et al., 2015; Yuan et al., 2015). Echinatin (Ech), a characteristic retrochalone present in glycyrrhiza, has been found to possess several pharmacological activities (Ji et al., 2016). For example, Ech promoted the apoptosis of esophageal squamous cell carcinoma cells via induction of reactive oxygen species (ROS) production or endoplasmic reticulum stress and activation of p38 mitogen-activated protein kinase/c-Jun N-terminal kinase signaling (Kwak et al., 2019). Furthermore, Ech treatment repressed epidermal growth factor receptor and mesenchymal-epithelial transition, induced ROS generation, and depolarized the mitochondria membrane potential, thereby inducing the apoptosis of non-small-cell lung cancer cells sensitive or resistant to gefitinib (Oh et al., 2020). Remarkably, the functional role of Ech in MI/R injury has not been well explored so far.

In this study, we aimed to characterize the functional role of Ech in the progression of MI/R injury, as well as elucidate the potential underlying molecular mechanisms. Our results demonstrated that Ech mitigated MI/R injury at least partially by suppressing Hippo/Yes kinase-associated protein (YAP) signaling, thus implying the protective effect of Ech on MI/R injury and providing an experimental basis for its clinical use.

## Materials and Methods

### *In Vivo* Model of Myocardial Ischemic/Reperfusion Injury

Male Wister rats (8 weeks old, weighing 250–280 g) from HuaFuKang (Beijing, China) were bred in laboratory settings and fed standard powdered rat diet and tap water. This study was approved by the Animal Care and use Committee of the Cangzhou Central Hospital. Rats were randomly divided into three groups: 1) Sham group, 2) MI/R group, and 3) MI/R + Ech group. After fasting overnight, all rats were operated following intraperitoneal injection of anesthesia with pentobarbitone (50 mg/kg). In the MI/R group, the heart was exposed by thoracic surgery. A 6-0 silk ligature was passed beneath the left anterior descending coronary artery (LAD). The successfully established MI rat model showed significant elevation of ST segment and presented a color change of myocardial tissue. After 30 min of occlusion, the LAD was reperfused for 24 h by opening the ligation. The same procedures were followed for the sham-operated rats, except the suture was left untied. The rats in the MI/R + Ech group were intraperitoneally injected with either vehicle or Ech (20, 40, and 80 mg/kg; purity ≥ 98%; Chengdu Must Biotechnology, Chengdu, China) 10 min before reperfusion. Ech was dissolved in DMSO and then diluted with phosphate buffer saline. The final concentration of DMSO was 1%, and the injection volume of each rat was 4 ml. During surgical procedures, the body temperature of rats was continuously monitored by a rectal thermometer. We kept the body temperature of rats at 37.0 ± 0.5 °C using a heating pad.

### Tetrazolium Chloride Staining

The animals were sacrificed after 24 h of reperfusion. Following this, the hearts were quickly resected, frozen in liquid nitrogen, and then cut into 2 mm thick sections. These sections were incubated with 1% TTC solution (Solarbio, Beijing, China) for 30 min away from the light. The infarcted tissues were white, while the viable tissues were red. Subsequently, these sections were fixed in 10% formalin, followed by analysis using ImageJ software (NIH, Rockville, MD, United States). The infarct size was calculated as the proportion of infarct area (white) to the total non-infract area (red).

### Evaluation of Lactate Dehydrogenase and Creatine Kinase-Myocardial Band Levels

At 24 h post reperfusion, blood sample collection was performed before the rats were sacrificed. The serum was collected after 30 min of blood clotting at 25 °C. The myocardium was also removed rapidly after the rats were sacrificed. The myocardium was washed, weighed, and then homogenized in phosphate buffered saline. Following centrifugation, the supernatants were carefully collected for all subsequent experiments. Next, the serum and supernatants were assayed for LDH and CK-MB levels using commercially-available enzyme-linked immunosorbent assay kits (Nanjing Jiancheng Bioengineering Institute, Nanjing, China) following the manufacturer’s instructions.

Myocardial damage was also evaluated by measuring LDH release using the LDH release Assay Kit (Beyotime, Shanghai, China). Briefly, H9c2 cells were harvested after Ech treatment, and then cultured with LDH release reagent for 1 h. Next, the supernatant was collected after centrifugation of H9c2 cells, followed by incubation with LDH detection reagent for 30 min in the dark. The release of LDH was analyzed by determination of the absorbance of each sample at 490 nm.

### Terminal Deoxynucleotidyl Transfer-Mediated dUTP Nick End-Labeling Assay

A One Step TUNEL Apoptosis Assay Kit (Beyotime) was utilized to evaluate cardiomyocyte apoptosis. In brief, tissue sections and cell smears were fixed for 30 min with 4% paraformaldehyde solution. After permeation with 0.3% Triton X-100, the samples were reacted with TUNEL detection solution for 1 h away from the light, following by staining with 4′,6-diamidino-2-phenylindole. Following sealing with antifade mounting medium, the samples were analyzed under a fluorescence microscope (Olympus, Tokyo, Japan). The apoptosis of cardiomyocyte was assessed by determining the ratio of the number of TUNEL-positive cells to the number of total cells.

### Determination of Caspase-3 Activity

Cell apoptosis was assessed by determination of caspase-3 activity using a caspase-3 activity assay kit (Beyotime), following the product manual. The myocardium and H9c2 cells were collected, lyzed, and centrifuged for 15 min, and the supernatants were collected. A Bradford Protein Assay kit (Bio-Rad, Hercules, CA, United States) was utilized to examine the concentration of extracted protein. The supernatants were reacted with Ac-DEVD-pNA for 1 h at 37 °C and the absorbance was measured at 405 nm.

### Quantitative Real-Time Polymerase Chain Reaction

RNA isolation was carried out using the TRIzol kit (Beyotime) as recommended by the manufacturer. cDNA synthesis was performed using PrimeScript™ RT Enzyme Mix I (Takara, Dalian, China). qRT-PCR analysis was performed using SYBR^®^-Green PCR Master Mix (Takara). The expression of YAP was analyzed by the 2^−ΔΔCT^ method using glyceraldehyde-3-phosphate dehydrogenase (GAPDH) as a housekeeping gene.

### Western Blotting

Total protein was isolated with radioimmunoprecipitation buffer and then quantified using the BCA protein assay kit (Solarbio), as per the manufacturer’s instructions. Cytoplasmic and nuclear proteins were isolated using the Nuclear and Cytoplasmic Protein Extraction Kit (Boster, Beijing, China) according to the manufacturer’s recommendations. After sodium lauryl sulfate-polyacrylamide gel electrophoresis, the isolated proteins were transferred onto polyvinylidene difluoride membranes. The membranes were blocked with 5% non-fat milk and then blotted with the following antibodies from Abcam (Cambridge, MA, United States) or Cell Signaling Technology (Beverly, MA, United States): anti-phosphorylated (p)-mammalian sterile 20-like protein kinase 1 (MST1) antibody (1:1,000), anti-p-large tumor suppressor 1 (LATS1) antibody (1:1,000), anti-p-YAP antibody (1:2,000), anti-YAP antibody (1:1,000), anti-H3 antibody (1:1,000), and anti-GAPDH antibody (1:3,000) at 4 °C overnight. GAPDH and histone H3 were used internal loading controls. Following 1 h of incubation with horseradish peroxidase-labeled secondary antibody (1:2,000; Abcam), blots were developed with an enhanced chemiluminescence kit (Beyotime) as per the manufacturer’s recommendations.

### *In Vitro* Model of Myocardial Ischemic/Reperfusion Injury

H9c2 cells were purchased from the Cell Bank of the Chinese Academy of Sciences (Shanghai, China) and then cultured in Dulbecco’s modified Eagle’s medium (DMEM) supplemented with 5% fetal bovine serum and 1% penicillin-streptomycin in a humidified 5% CO_2_ incubator at 37 °C. To establish an *in vitro* model of MI/R injury, H9c2 cells were cultured for 4 h in glucose- and serum-free medium under 95% N_2_/5% CO_2_ at 37 °C. When the oxygen content is more than 1%, N_2_ will be automatically charged into the incubator to always maintain the oxygen concentration of the incubator at the set value. Next, cells were cultured for 24 h in DMEM plus Ech (10 or 20 μm) at 37 °C in a humidified incubator with 5% CO_2_ and 95% air. H9c2 cells were then harvested for subsequent experiments.

Small interfering RNA specific for YAP (si-YAP) and si-negative control (NC) were purchased from GenePharma (Shanghai, China). Cell transfection was performed using Lipofectamine™ 2000 (Invitrogen, Carlsbad, CA, United States) as per the instruction manual. The knockdown efficiency was affirmed using qRT-PCR and western blot analyses.

### Cell Counting Kit-8 Assay

H9c2 cells were seeded into a 96-well plate and cultured overnight in a humidified incubator at 37 °C with 5% CO_2_. Following this, cells were exposed to different doses (0, 1, 5, 10, 20, 40, and 80 μm) of Ech for 24 h. Following the addition of CCK-8 solution (Solarbio), H9c2 cells were grown in a 5% CO_2_ incubator at 37 °C for 2 h. The absorbance of each sample at 450 nm was analyzed using a microplate reader.

### Statistical Analysis

All results are presented as mean ± standard deviation of three independent experiments. Statistical analysis was performed using one-way analysis of variance with post-hoc Bonferroni correction using SPSS 20.0 software (SPSS Inc., Chicago, IL, United States). Statistical significance was set at *p* < 0.05.

## Results

### Ech Mitigates I/R-Induced Myocardial Injury *In Vivo*

**FIGURE 1:**
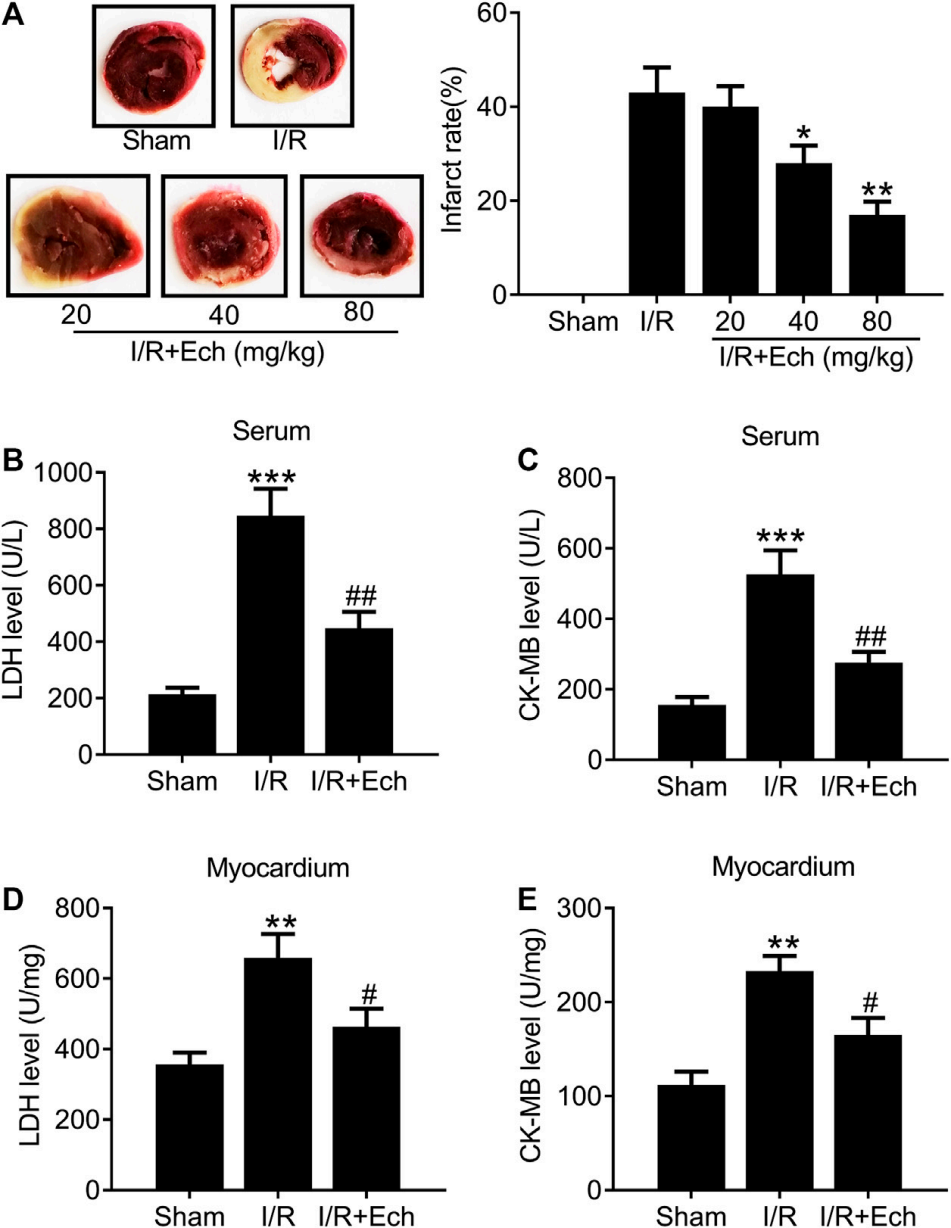
Ech mitigates I/R-induced myocardial injury *in vivo*. MI/R rats were subjected to Ech (20, 40, and 80 mg/kg) treatment before reperfusion. **(A)** Representative heart sections stained with TTC after MI/R and Ech administration. Myocardial infarction was measured as the proportion of infarct area (white) to the total non-infract area (red) (*n* = 5). **(B, C)** The degree of myocardial injury was assessed by measurement of serum LDH and CK-MB levels (*n* = 5). **(D, E)** Measurements of myocardial LDH and CK-MB levels in the heart of rats (*n* = 5). *p < 0.05 and **p < 0.01 compared with MI/R rats.

To examine the protective effect of Ech on MI/R injury, MI/R rats were subjected to Ech (20, 40, and 80 mg/kg) treatment before reperfusion. After 24 h of reperfusion, the myocardial infarct size was measured. As shown in Figure 1A, MI/R induced myocardial infarction relative to the sham group. However, Ech administration markedly reduced MI/R-induced myocardial infarction. Considering the significant cardioprotective effect of Ech at 80 mg/kg, we chose this concentration for all following experiments. MI/R rats also showed a significant increase in the levels of serum LDH and CK-MB relative to the sham group; however, Ech treatment attenuated MI/R-induced elevation of LDH and CK-MB serum levels (Figures 1B,C). Similarly, MI/R increased the levels of myocardial LDH and CK-MB in the heart of rats, which was inhibited by Ech treatment (Figures 1D,E).

### Ech Inhibits Cardiomyocyte Apoptosis in Myocardial Ischemic/Reperfusion Rats

**FIGURE 2:**
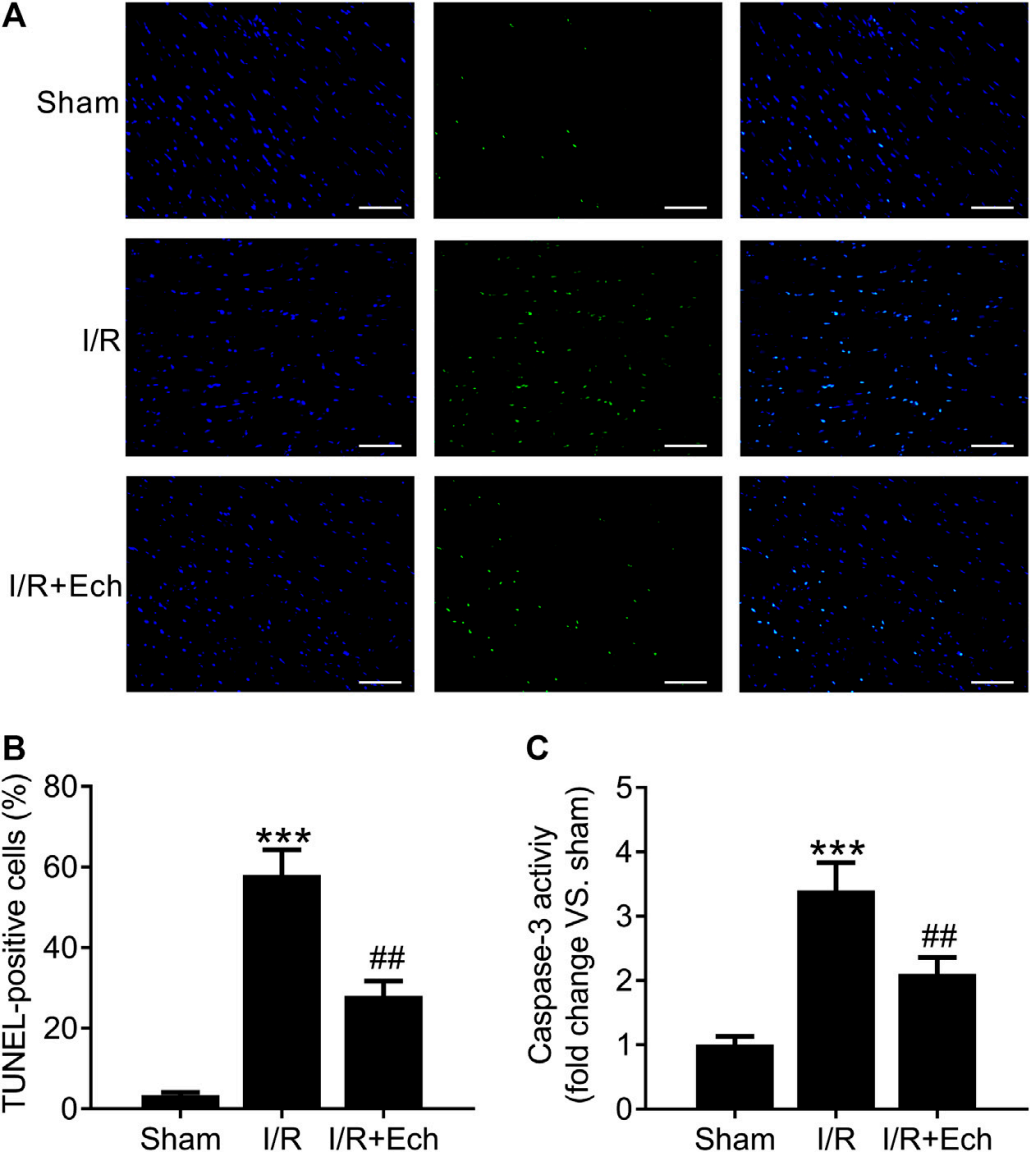
Ech inhibits cardiomyocyte apoptosis in MI/R rats. **(A)** Representative images and **(B)** quantification of TUNEL staining indicated that Ech administration mitigated MI/R-induced cardiomyocyte apoptosis (*n* = 4). **(C)** Analysis of caspase-3 activity showed that the increase in caspase-3 activity in MI/R rats was reversed by Ech treatment (*n* = 4). ***p < 0.001 compared with the sham group; ^##^ *p* < 0.01 compared with MI/R rats.

Next, we evaluated the cardioprotective effect of Ech against MI/R-induced cardiomyocyte apoptosis. Compared to sham-operated rats, MI/R rats showed an increase in the number of TUNEL-positive cells, as determined by TUNEL assay. However, Ech treatment significantly inhibited MI/R-induced cardiomyocyte apoptosis (Figures 2A,B). To clarify the mechanism by which Ech protects against MI/R-induced cardiomyocyte apoptosis, the effect of Ech treatment on caspase-3 activity was further examined in the cardiac tissues of MI/R rats. Results showed that MI/R significantly enhanced caspase-3 activity; however, this effect was blocked by Ech treatment (Figure 2C).

### Ech Inhibits the Hippo/YAP Signaling Pathway *In Vivo*

**FIGURE 3:**
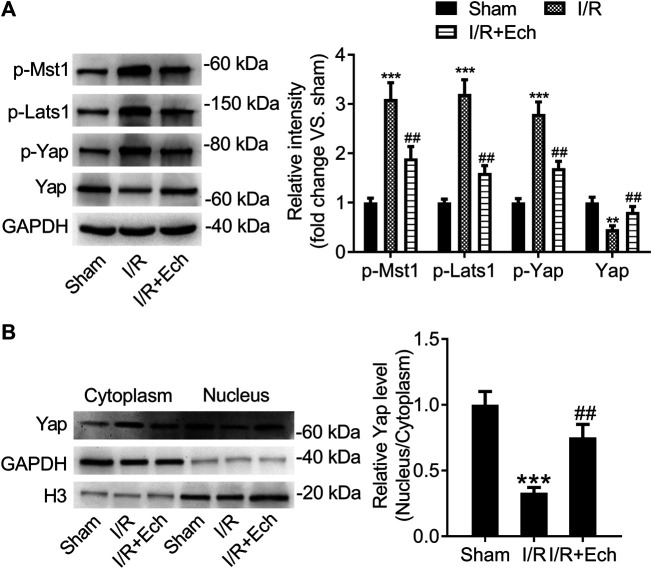
Ech inhibits the Hippo/YAP signaling pathway *in vivo*. **(A)** Representative images of western blot analysis and quantification of p-MST1, p-LATS1, p-YAP, and YAP expression in the cardiac tissues of MI/R rats (*n* = 4). **(B)** Representative images of western blot analysis and quantification of cytoplasmic and nuclear YAP expression in the cardiac tissues of MI/R rats (*n* = 4). **p < 0.01 and ***p < 0.001 compared with the sham group; ^##^ *p* < 0.01 compared with MI/R rats.

To explore whether the Hippo/YAP signaling pathway mediates the protective effect of Ech on MI/R injury, we detected the protein expression levels of p-MST1, p-LATS1, p-YAP, and YAP using western blotting. Results showed that the protein levels of p-MST1, p-LATS1, and p-YAP were elevated, but those of YAP were reduced in the cardiac tissues of MI/R rats compared to sham-operated rats. However, administration of Ech significantly reduced the levels of p-MST1, p-LATS1, and p-YAP and increased the levels of YAP relative to the MI/R group (Figure 3A). Besides, our results demonstrated that MI/R restrained YAP nuclear translocation, which was reversed by Ech treatment. This was also affirmed by obviously lower nuclear to cytoplasmic ratio of YAP protein level in MI/R rats compared to sham-operated rats, and markedly higher nuclear to cytoplasmic ratio of YAP protein level in Ech-treated MI/R rats relative to MI/R rats (Figure 3B).

### Ech Protects H9c2 Cells Against Hypoxia/Reoxygenation-Induced Myocardial Damage

**FIGURE 4:**
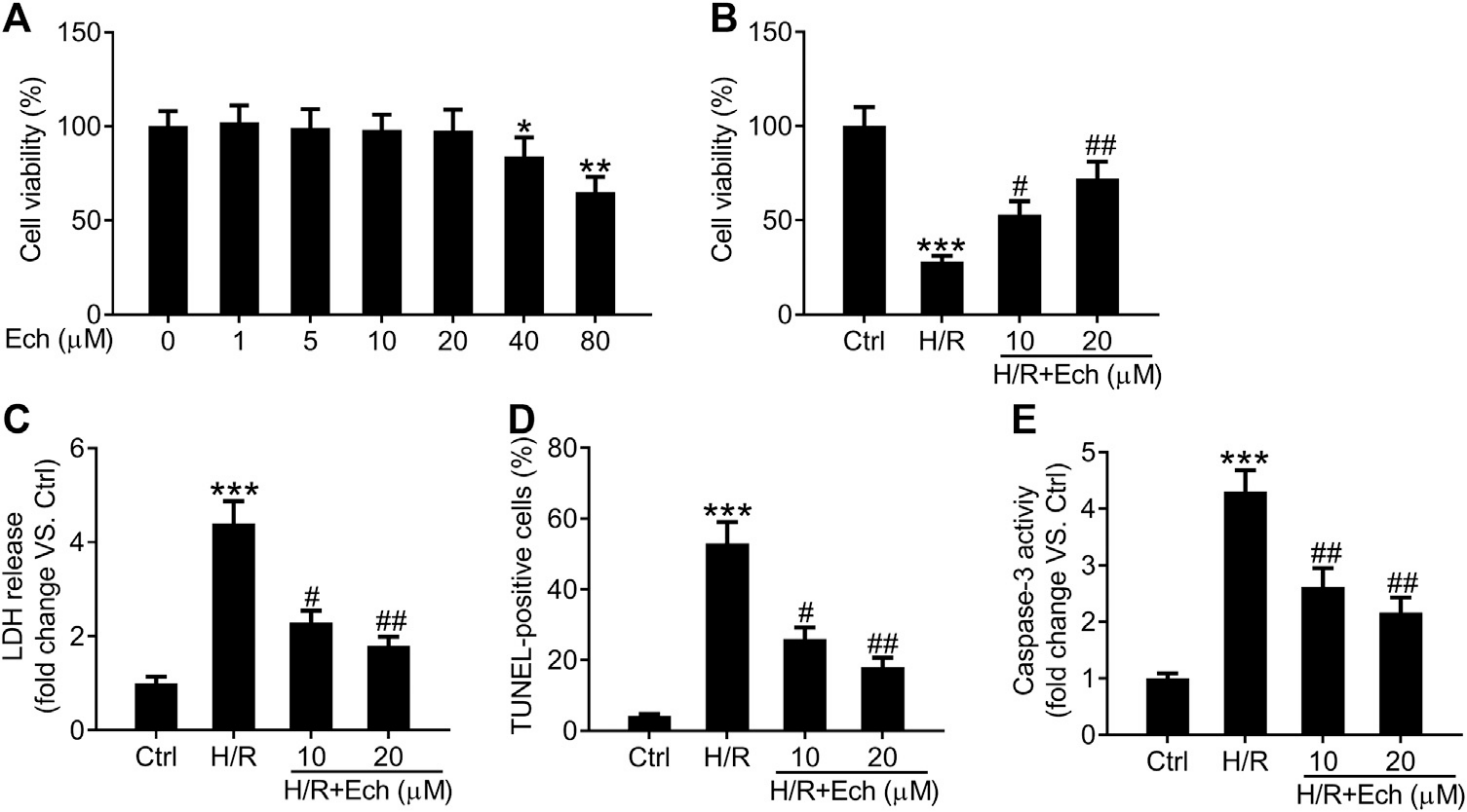
Ech protects H9c2 cells against H/R-induced injury. H9c2 cells were exposed to increasing doses (0, 1, 5, 10, 20, 40, and 80 μm) of Ech. **(A)** The cytotoxicity of Ech in H9c2 cells was evaluated using CCK-8 assay. H9c2 cells were exposed to H/R and then treated with Ech (10 and 20 μm), (*n* = 3). **(B)** CCK-8 assay results showing the protective effect of Ech on H/R-induced inhibition of H9c2 cell viability (*n* = 3). **(C)** Results showing the inhibitory effect of Ech on H/R-induced LDH release (*n* = 3). **(D)** Representative images and quantification of TUNEL staining showing the protective effect of Ech on H/R-induced H9c2 cell apoptosis (*n* = 3). **(E)** Analysis of caspase-3 activity showing the reduced activity of caspase-3 in H9c2 cells treated with H/R and Ech relative to cells treated with H/R only (*n* = 3). *p < 0.05, **p < 0.01, and ***p < 0.001 compared with the control (Ctrl) group; ^#^ *p* < 0.05 and ^##^ *p* < 0.01 compared with the H/R group.

To further confirm the cardioprotective effect of Ech on MI/R injury, we established an *in vitro* model of MI/R injury by exposing H9c2 cells to H/R. As determined by CCK-8 assay, 40 and 80 μm of Ech markedly restrained the viability of H9c2 cells compared to the controls, but 1, 5, 10, and 20 μm of Ech did not influence H9c2 cell viability (Figure 4A). Therefore, 10 and 20 μm of Ech were selected for subsequent experiments. H9c2 cells subjected to H/R condition showed a marked decline in cell viability; however, this effect was blocked by treatment with 10 and 20 μm Ech (Figure 4B). Consistently, a marked elevation in LDH release was observed in H9c2 cells exposed to H/R, and this effect was abolished following Ech (10 and 20 μm) treatment (Figure 4C). In parallel, H/R promoted H9c2 cell apoptosis, as indicated by the increased number of TUNEL-positive cells. However, Ech at concentrations of 10 and 20 μm attenuated H/R-induced cell apoptosis (Figure 4D). Moreover, H/R exposure increased caspase-3 activity in H9c2 cells, and this effect was counteracted by Ech administration (Figure 4E).

### Ech Restrains Phosphorylation of MST1 and LATS1, and Triggers YAP Nuclear Translocation *In Vitro*

**FIGURE 5:**
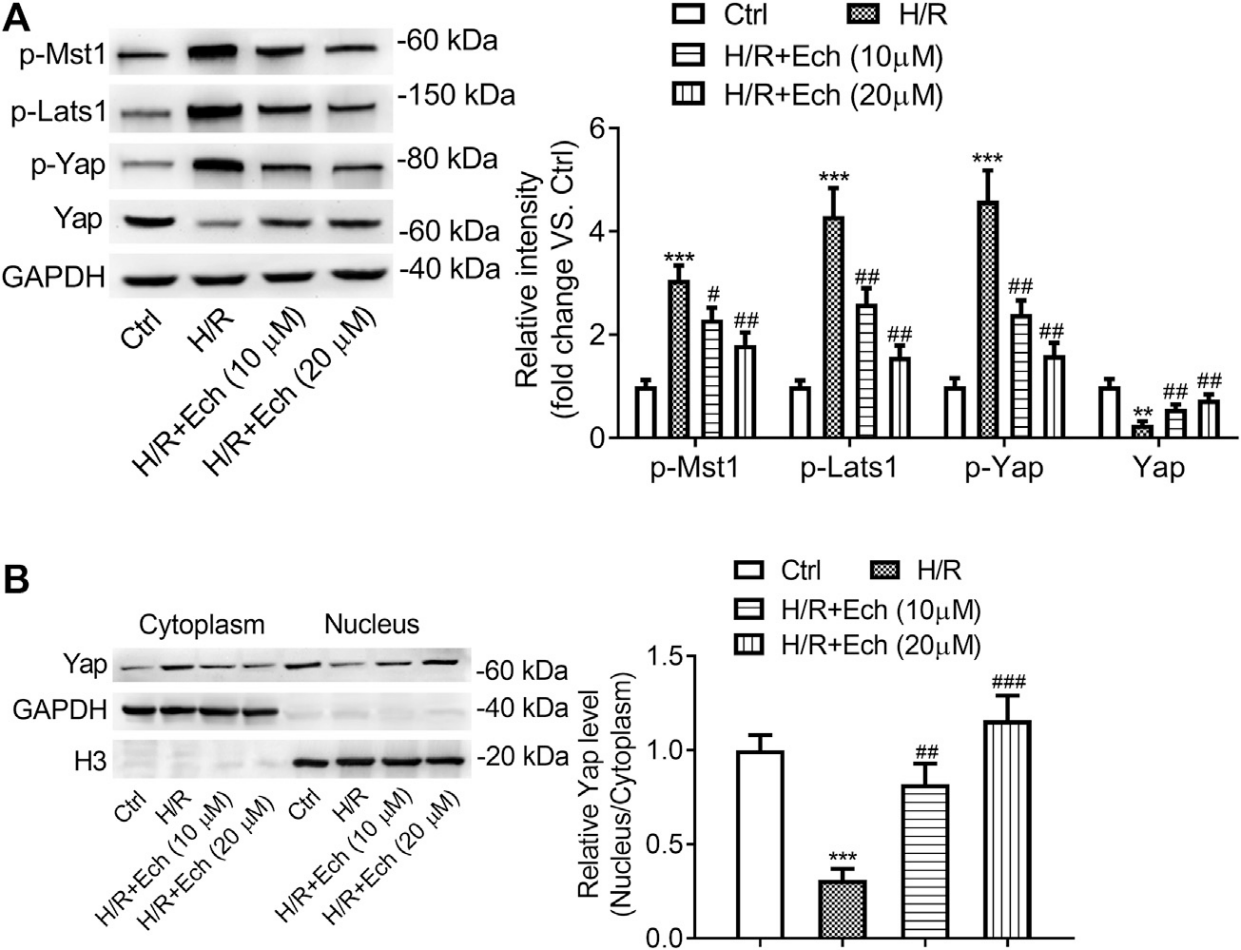
Ech restrains phosphorylation of MST1 and LATS1, and triggers YAP nuclear translocation *in vitro*. H9c2 cells were subjected to H/R and then treated with Ech (10 and 20 μm). **(A)** Representative images of western blot analysis and quantification of p-MST1, p-LATS1, p-YAP, and YAP expression in H9c2 cells (*n* = 3). **(B)** Representative images of western blot analysis and quantification of cytoplasmic and nuclear YAP expression in H9c2 cells (*n* = 3). **p < 0.01 and ***p < 0.001 compared with the Ctrl group; ^#^ *p* < 0.05, ^##^ *p* < 0.01, and ^###^ *p* < 0.001 compared with the H/R group.

To determine the impact of Ech on Hippo/YAP signaling *in vitro*, H9c2 cells were exposed to H/R, followed by treatment with Ech (10 and 20 μm). As determined by western blot analysis, the expression level of p-MST1, p-LATS1, and p-YAP was elevated, but that of YAP was reduced in H/R-treated H9c2 cells; however, these effects were abolished by administration of Ech (Figure 5A). In addition, our results showed that exposure to H/R inhibited YAP nuclear translocation, as evidenced by a decrease in the nuclear to cytoplasmic ratio of YAP protein. However, this effect was blocked by Ech administration (Figure 5B).

### Loss of YAP Counteracts the Protective Effect of Ech on Hypoxia/Reoxygenation-Induced Myocardial Cell Injury *In Vitro*

**FIGURE 6:**
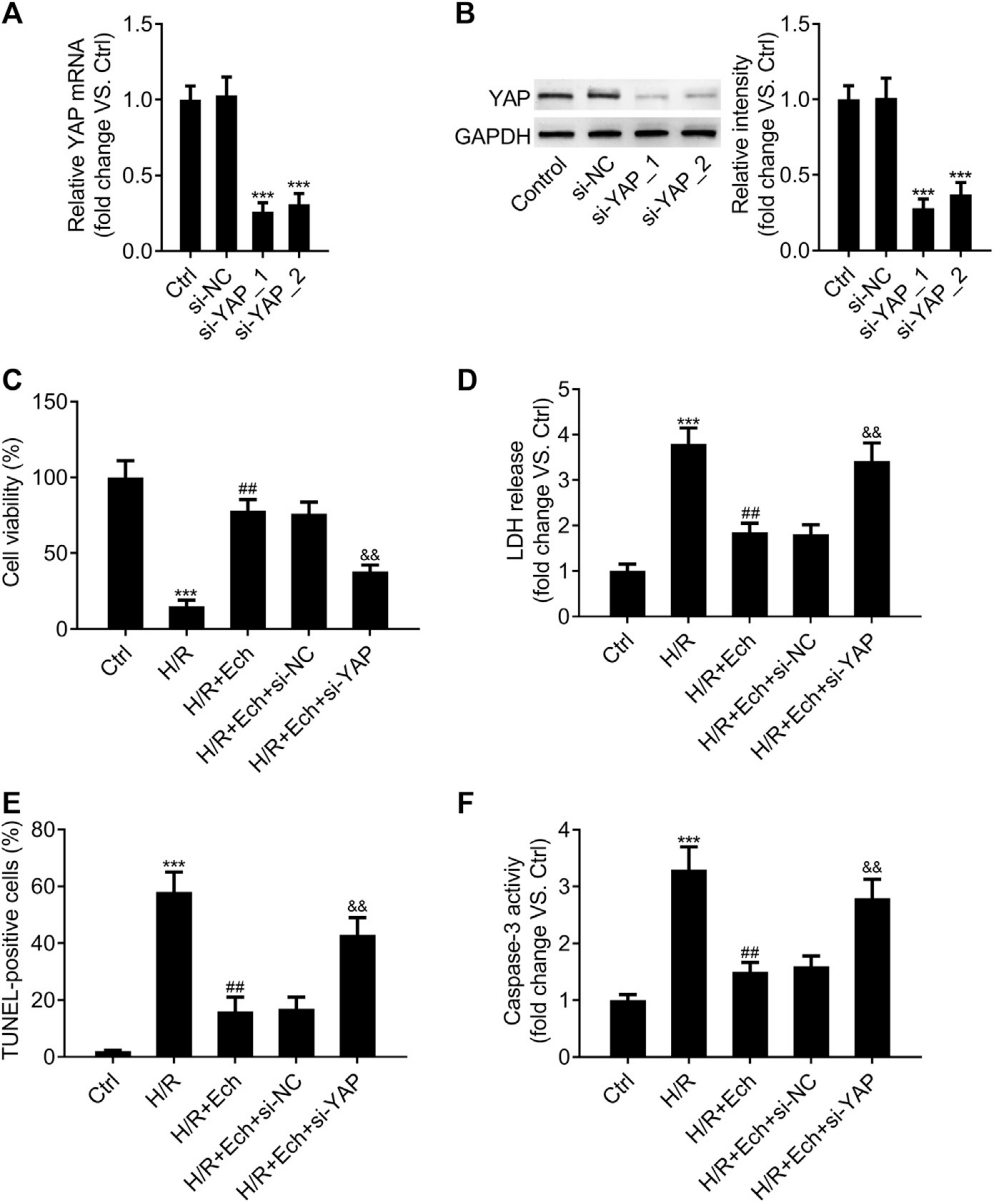
Loss of YAP counteracts the protective effect of Ech on H/R-induced cell injury *in vitro*. **(A, B)** H9c2 cells were transfected with si-YAP and si-NC, and the silencing efficiency was determined by qRT-PCR and western blot analyses. After exposure to H/R, H9c2 cells were treated with Ech (10 μm) for 24 h (*n* = 3). **(C)** Results of CCK-8 assay showed that silencing of YAP abrogated the protective effect of Ech on H/R-induced cell viability inhibition (*n* = 3). **(D)** The inhibitory effect of Ech on H/R-induced LDH release was blocked by YAP knockdown (*n* = 3). **(E)** Representative images and quantification of TUNEL staining showing that the protective effect of Ech on H/R-induced H9c2 cell apoptosis was counteracted by si-YAP transfection (*n* = 3). **(F)** Analysis of caspase-3 activity showing the increased activity of caspase-3 in H9c2 cells treated with si-YAP, H/R, and Ech relative to H9c2 cells treated with H/R and Ech (*n* = 3). ***p < 0.001 compared with the si-NC and Ctrl groups; ^##^ *p* < 0.01 compared with the H/R group; ^&&^ *p* < 0.01 compared with the H/R + Ech + si-NC group.

In order to determine whether silencing of YAP counteracts the protective effect of Ech on H/R-induced myocardial cell injury *in vitro*, si-YAP and si-NC were transfected into H9c2 cells and the silencing efficiency was evaluated by qRT-PCR assay. As expected, YAP expression was much lower in the si-YAP group than in the si-NC group (Figure 6A). Western blot analysis showed similar results (Figure 6B). Moreover, we found that silencing of YAP could abrogate the protective effect of Ech on H/R-induced inhibition of H9c2 cell viability (Figure 6C). Knockdown of YAP also abolished Ech-mediated inhibition of LDH release in H9c2 cells exposed to H/R (Figure 6D). Furthermore, the results of TUNEL assay revealed that the protective effect of Ech on H/R-induced H9c2 cell apoptosis was hindered following si-YAP transfection, as indicated by the increase in number of TUNEL-positive cells (Figure 6E). Likewise, Ech treatment decreased caspase-3 activity in H/R-induced H9c2 cells, and this effect was counteracted by YAP silencing (Figure 6F).

## Discussion

The mechanism of MI/R injury is complex and involves multiple biological processes, including cardiomyocyte loss, which plays a vital role (Wu et al., 2018). Myocardial ischemia initiates multiple deleterious cascades, such as oxidative stress, autophagy, and inflammatory response, and then aggravates cardiomyocyte injury, contributing to contractile dysfunction and apoptosis of cardiomyocytes (Granger and Kvietys, 2015; Kurian et al., 2016). Damage of mitochondrial membrane integrity causes release of cytochrome c, which in turn triggers the activation of caspase-3 and finally results in cardiomyocyte apoptosis (Badalzadeh et al., 2015). It is known that reduction of cardiomyocyte apoptosis is a crucial factor in improving myocyte contractile function.

Recently, there is emerging interest in the use of Ech because of its antioxidant and anti-inflammatory potential. The use of Ech as a natural antioxidant has been well established previously. Ech was shown to exert its antioxidant activity via an electron transfer and a proton transfer in aqueous solution and through the hydrogen atom transfer pathway in organic solution (Liang et al., 2018). Additionally, the anti-inflammatory effect of Ech has been also demonstrated. Ech treatment was shown to block lipopolysaccharide-induced elevation of prostaglandin E2 and interleukin-6 levels in RAW 264.7 macrophages (Fu et al., 2013). Furthermore, Ech functioned as an activator of nuclear factor erythroid 2-related factor 2 to mitigate hepatoxicity and carbon tetrachloride-induced acute liver injury in mice, suggesting its antioxidant potential (Lin et al., 2017). Moreover, Ech reduced the levels of LDH and CK-MB, as well as the content of malondialdehyde, interleukin-6, and tumor necrosis factor-α, and upregulated the activity of superoxide dismutase, thereby reducing myocardial infarct size and cardiomyocyte apoptosis (Tian et al., 2016). In the present study, our results showed that Ech administration mitigated MI/R-induced myocardial infarction and myocardial apoptosis *in vivo* and *in vitro*, thus confirming the cardioprotective effect of Ech. Our findings support the notion of Ech as a promising novel agent to remit MI/R injury.

MI/R is a complicated biological process, which is mediated by several signaling mechanisms. To further characterize the protective effect of Ech on MI/R injury, we investigated the potential signaling mechanisms underlying the action of Ech on cardiomyocytes. Several lines of evidence suggest that Hippo/YAP signaling plays a vital role in various biological processes, including cardiovascular development, cardiomyocyte regeneration, and autophagy (Plouffe et al., 2015). When the Hippo pathway is “switched on,” MST1/2 is activated by adaptor protein salvador 1-induced phosphorylation, which in turn phosphorylates and activates LATS1/2 (Mia and Singh, 2019). Activated LATS1/2 directly phosphorylate YAP, the core effector of Hippo signaling, followed by its cytoplasmic retention and then degradation via the proteosomal pathway (Kim and Jho, 2018). Conversely, non-phosphorylated YAP accumulates in the nucleus and functions as a transcriptional cofactor to participate in various cellular processes when Hippo signaling is “switched off” (Windmueller and Morrisey, 2015). Recently, dysregulation of Hippo/YAP signaling has been shown to be involved in the development of MI/R injury (Zhang and Del Re, 2017). For example, in a previous study, neurofibromin-2 activated by oxidative stress promoted cardiomyocyte apoptosis, induced the activation of MST1 and suppression of YAP, as well as protected against cardiomyocyte I/R injury, strongly suggesting a key role of Hippo signaling in MI/R injury (Matsuda et al., 2016). Decreased expression of MST1, LATS1, and YAP/YAZ was also reported in AC16 cells subjected to H/R. Moreover, upregulation of YAP1 attenuated MI/R damage in AC16 human cardiomyocytes by co-activation of the Wnt/β-catenin pathway (Khan et al., 2019). Additionally, blockage of the Hippo/YAP signaling pathway diminished the protective effect of melatonin on MI/R-induced cardiomyocyte death and mitochondrial damage, indicating the involvement of Hippo/YAP signaling in MI/R injury (Ma and Dong, 2019). Furthermore, activation of Hippo could antagonize YAP-FoxO1 to restrain the expression of catalase and MnSOD, and then enhance oxidative stress-induced cell death during MI/R, suggesting that Hippo/YAP signaling mediates oxidative stress-induced MI/R damage (Shao et al., 2014). This finding highlights that targeting the Hippo/YAP pathway is regarded as a potential approach to reduce MI/R injury Mia et al. (2017). However, whether Hippo signaling mediates the protective effect of Ech on MI/R injury remains poorly understood. In this study, our results showed that Ech treatment inhibited the activation of Hippo/YAP signaling and induced the nuclear translocation of YAP, suggesting a strong correlation between Hippo/YAP signaling and MI/R injury. Moreover, silencing of YAP counteracted the protective effect of Ech on H/R-induced myocardial cell injury, thus providing evidence that Ech protects against MI/R damage at least partially by inducing Hippo/YAP pathway inactivation.

As a limitation of this study, YAP silencing was conducted only in *in vitro* experiments. We did not explore whether inhibition of Hippo/YAP signaling pathway can counteract the cardioprotective effect of Ech on MI/R injury *in vivo*. In our future work, we will identify whether other mechanisms are also involved in the cardioprotective effect of Ech on MI/R injury.

## Conclusion

Overall, our data revealed that Ech administration attenuated myocardial infarction and repressed cardiomyocyte apoptosis in *in vivo* and *in vitro* models of MI/R injury. Subsequent mechanistic experiments demonstrated that Ech protected against MI/R injury at least partially by suppressing the activation of Hippo/YAP signaling. Furthermore, we revealed the cardioprotective role and mechanism of action of Ech in MI/R injury, thus providing an experimental basis for its clinical use.

## Data Availability Statement

The raw data supporting the conclusions of this article will be made available by the authors, without undue reservation.

## Ethics Statement

The animal study was reviewed and approved by the Animal Care and Use Committee of the Cangzhou Central Hospital.

## Author Contributions

JN, YnL, and XS performed the main experiments. YuL and YiL performed statistical analysis of all data. YaL contributed to conception, supervision, administration, and validation of this project. All authors reviewed the manuscript.

## Conflict of Interest

The authors declare that the research was conducted in the absence of any commercial or financial relationships that could be construed as a potential conflict of interest.
